# Bacteriota and Antibiotic Resistance in Spiders

**DOI:** 10.3390/insects13080680

**Published:** 2022-07-27

**Authors:** Miroslava Kačániová, Margarita Terentjeva, Przemysław Łukasz Kowalczewski, Mária Babošová, Jana Ivanič Porhajašová, Wafaa M. Hikal, Mariia Fedoriak

**Affiliations:** 1 Institute of Horticulture, Faculty of Horticulture and Landscape Engineering, Slovak University of Agriculture, Tr. A. Hlinku 2, 94976 Nitra, Slovakia; 2 Department of Bioenergy, Food Technology and Microbiology, Institute of Food Technology and Nutrition, University of Rzeszow, 4 Zelwerowicza St., 35-601 Rzeszow, Poland; 3 Institute of Food and Environmental Hygiene, Faculty of Veterinary Medicine, Latvia University of Life Sciences and Technologies, LV-3004 Jelgava, Latvia; margarita.terentjeva@llu.lv; 4 Department of Food Technology of Plant Origin, Poznań University of Life Sciences, 31 Wojska Polskiego St., 60-624 Poznań, Poland; przemyslaw.kowalczewski@up.poznan.pl; 5 Institute of Plant and Environmental Sciences, Faculty of Agrobiology and Food Resources, Slovak University of Agriculture, Tr. A. Hlinku 2, 94976 Nitra, Slovakia; maria.babosova@uniag.sk (M.B.); jana.porhajasova@uniag.sk (J.I.P.); 6 Department of Biology, Faculty of Science, University of Tabuk, P.O. Box 741, Tabuk 71491, Saudi Arabia; wafaahikal@gmail.com; 7 Environmental Parasitology Laboratory, Water Pollution Research Department, Environment and Climate Change Institute, National Research Centre (NRC), 33 El–Behouth St., Dokki, Giza 12622, Egypt; 8 Department of Ecology and Biomonitoring, Institute of Biology, Chemistry and Bioresources, Yuriy Fedkovych Chernivtsi National University, 2 Kotsyubynskyi Street, 58012 Chernivtsi, Ukraine; m.m.fedoriak@gmail.com

**Keywords:** spiders, exogenes microbiota, mass spectrometry, antibiotic resistance

## Abstract

**Simple Summary:**

The microbiomes of insects are known for having a great impact on their physiological properties for survival, such as nutrition, behavior, and health. In nature, spiders are one of the main insect predators, and their microbiomes have remained unclear yet. It is important to explore the microbiomes of spiders with the positive effect in the wild to gain an insight into the host–bacterial relationship. The insects have been the primary focus of microbiome studies from all arthropods. Although the research focused on the microbiome of spiders is still scarce, there is a possibility that spiders host diverse assemblages of bacteria, and some of them alter their physiology and behavior. According to our findings, there is a need for holistic microbiome studies across many organisms, which would increase our knowledge of the diversity and evolution of symbiotic relationships. Antimicrobial resistance is one of the most serious global public health threats in this century. Therefore, the knowledge and some information about insects and their ability to act as reservoirs of antibiotic-resistant microorganisms should be determined in order to ensure that they are not transferred to humans. It is important to monitor the microbiome of spiders found in human houses and the transmission of resistant microorganisms, which can be dangerous in relation to human health.

**Abstract:**

Arthropods are reported to serve as vectors of transmission of pathogenic microorganisms to humans, animals, and the environment. The aims of our study were (i) to identify the external bacteriota of spiders inhabiting a chicken farm and slaughterhouse and (ii) to detect antimicrobial resistance of the isolates. In total, 102 spiders of 14 species were collected from a chicken farm, slaughterhouse, and buildings located in west Slovakia in 2017. Samples were diluted in peptone buffered water, and Tryptone Soya Agar (TSA), Triple Sugar Agar (TSI), Blood Agar (BA), and Anaerobic Agar (AA) were used for inoculation. A total of 28 genera and 56 microbial species were isolated from the samples. The most abundant species were *Bacillus pumilus* (28 isolates) and *B. thuringensis* (28 isolates). The least isolated species were *Rhodotorula mucilaginosa* (one isolate), *Kocuria rhizophila* (two isolates), *Paenibacillus polymyxa* (two isolates), and *Staphylococcus equorum* (two isolates). There were differences in microbial composition between the samples originating from the slaughterhouse, chicken farm, and buildings. The majority of the bacterial isolates resistant to antibiotics were isolated from the chicken farm. The isolation of potentially pathogenic bacteria such as *Salmonella*, *Escherichia*, and *Salmonella* spp., which possess multiple drug resistance, is of public health concern.

## 1. Introduction

Plants and animals are inhabited by specific microbial communities, which form specific ecosystems strongly associated with their hosts. Those communities function as diverse ecosystems where the interactions between the microbiota and their hosts are of importance [1,2,3,4]. These phenomena have been referred to as hologenomic adaptations, and microorganisms have been developing new properties as a result of the symbiosis between the microorganisms and the hosts [5,6,7].

The symbiotic bacteria were found to be gut-associated and were identified in the intestinal lumen or crypts where they participate in digestion by providing their host with nutrients. The ectosymbiotic bacteria may be present in mycangia or attached to the body surface and were found to fulfill the immunity functions. The gut microbiomes of insects were known to have a great impact on their physiological properties for survival, such as nutrition, behavior, and health. In nature, spiders are one of the main predators of insects, and yet their gut microbiomes remain unclear. It is important to explore the gut microbiomes of spiders in the wild to gain an insight into the host–bacterial relationship [8,9,10,11,12,13].

Spiders (Araneae) are the most common terrestrial predators and natural enemies of insects, with some of them being of agricultural importance as a part of biological pest control [14,15]. Previous studies have mostly focused on the symbionts and their impact on the spiders’ reproduction, while other studies evaluated the effects of social spider microbiota on their evolution [16]. Therefore, limited information on the bacteria inhabiting the external surface of the spider is available.

Microbiota of spiders has been associated with relatively low genetic diversity, and *Chlamydiales*, *Borrelia*, and *Mycoplasma* were the most abundant symbionts of social spiders [16]. High incidence of symbiotic *Wolbachia*, *Rickettsia*, *Cardinium*, and *Spiroplasma* in spiders were described previously [17,18,19]. Phylum Proteobacteria was dominant in the gut microbiota of three spider species, with Burkholderia being among the most abundant. Tenericutes, Actinobacteria, Firmicutes, Acidobacteria, and Bacteroidetes were found to inhabit the gut without particular reference to the feeding habits of spiders [20].

While the presence of symbiotic microorganisms in insects may significantly differ between species, environmental microorganisms may be occasionally isolated from spiders with subsequent contamination of body cavities. The presence of *Staphylococcus* spp. in body swaps and *Staphylococcus aureus* in excreta samples was identified in microbiota studies of *Rabidosa rabida* [21]. The presence of opportunistic pathogens such as *Morganella*, *Providencia*, Proteus, or Acinetobacter in insects indicates that spiders also may serve as a potential vector of different pathogens important for animal, human, and environmental health [22,23,24]. There are limited studies on the prevalence of potentially pathogenic microorganisms on studies, whilst spiders are among the frequent habitants of different premises. The role of insects in the transfer of different pathogens has been documented [25]. Therefore, studies on the exobacteriome are needed to explore the possible importance of spiders on the transmission of different microorganisms are needed.

Antimicrobial resistance is the main public health threat with human, animal, and environmental health affected. Antimicrobials and their residues may spread into the environment after application in humans or animals with contamination of different terrestrial and aquatic habitats [26]. Antibiotic resistance genes were found in the collembolan microbiome that has been linked to the presence of arthropod [27]. The ecology and chemistry of soil have been changing significantly as a response to the land use changes that possess an impact on the insects and their associated microbiome [28]. Since the microbiome of the arthropod may affect the nutrient cycle within the ecosystem by possibly being the carriers of antimicrobial resistance genes, there is a need to study the microbiota of the arthropod and its antimicrobial resistance.

The aim of this study was to study external bacteriota of spiders from the slaughterhouse, chicken farm, and buildings and to detect the antimicrobial resistance of isolated microorganisms.

## 2. Materials and Methods

### 2.1. Sample Preparation

A total of 102 spiders of 14 species were sampled in the present research from the slaughterhouse, buildings, and chicken farms in 2017 (Table 1).

The spiders were visually identified by microscopy. All spiders were all identified as nonendangered and nonprotected species (Table 1). The collected spiders were frozen at −20 °C for 1 min. A sample of external surfaces of each spider was obtained by transferring the spider into a sterile 2 mL micro centrifuge tube, and 1 mL of sterile 0.87% (*w/v*) NaCl was added. Then, a 100 µL of the sample was plated onto agars for detection of different bacterial groups.

Tripton Soya agar (TSA), Tripton Sugar Iron agar (TSI), Anaerobic agar (AA), and Blood agar (BA) supplemented with 7% of horse blood (Sigma-Aldrich^®^, St. Louis, MO, USA) were used for detection of the total microbial count, Enterobacteriales, anaerobic and fastidious microorganisms, respectively. Inoculated TSA was incubated at 30 °C for 24–48 h, TSI agar at 37 °C for 18–24 h and AA at 30 °C for 24–48 h and BA at 37 °C for 24–48 h. AA was incubated anaerobically while all other agars aerobically. After the assessment of microbial growth, eight bacterial colonies with different macroscopic characteristics were selected from each agar for species confirmation. Isolates were subcultured on TSA at 37 °C for 24 h and used for MALDI-TOF identification.

### 2.2. Identification of Microbiota

Identification of microbiota was performed with MALDI-TOF MS Biotyper (Bruker Daltoncs, Bremen, Germany). Samples were prepared for investigation according to MALDI TOF MS Biotyper manufacturer’s protocol. The bacterial suspension was prepared into 300 μL of distilled water and 900 µL and centrifuged for 2 min at 14,000 rpm. After the supernatant was discarded, centrifugation was repeated by adding 10 µL of 70% formic acid and 10 μL of acetonitrile were added to the pellet, which was centrifuged for 2 min at 14,000 rpm. Then, 1 μL of the supernatant was used for investigation, and the suspension was covered with a matrix, α-Cyano-4-hydroxycinnamic acid, in a volume of 1 μL. Identification was performed with Microflex LT (Bruker Daltonics, Bremen, Germany) instrument and Flex Control 3.4 software, and Biotyper Realtime Classification 3.1 with BC-specific software. Confidence scores of ≥2.0 and ≥1.7 were applied for identification at species and genus level, respectively.

### 2.3. Antimicrobial Resistance Testing

Antimicrobial susceptibility tests were detected by the disc diffusion method. Each isolated microbial species from each spider was tested for antibiotic resistance according to the EUCAST (2022). Antimicrobial resistance against imipenem (IPM), meropenem (MEM), ciprofloxacin (CIP), vancomycin (VA), linezolid (LZD), tobramycin (TOB), tigecycline (TGC), amikacin (AK), norfloxacin (NOR), tetracycline (TE), and rifampicin (RD) (Oxoid, Basingstoke, UK) was examined. The antimicrobial resistance testing results were evaluated in accordance with the EUCAST [29].

For detection of antimicrobial resistance, bacterial isolates were cultured in Muller Hinton broth (Sigma-Aldrich^®^, St. Louis, MO, USA) for at 37 °C 24 h and yeast in Sabouraud broth (Sigma-Aldrich^®^, St. Louis, MO, USA) at 25 °C for 24 h. After incubation, the microbial suspensions in sterile distilled water of concentration 10^5^ cells/mL (A620 nm = 0.388, equivalent to a McFarland standard) were used for testing. Three replicates were tested for each isolated strain.

### 2.4. Statistical Analyses

Data analysis was conducted using R. For microbial counts, the mean and standard deviation (SD) were calculated, and *t*-test was used for calculation of significance of differences between the microbial counts in different spider species. *p*-values for evaluation of the significance of the results were *p* ≤ 0.05, *p* ≤ 0.01, and *p* ≤ 0.001.

## 3. Results

### 3.1. Qualitative Analysis of Isolated Microbiota from Spiders

The microbial counts identified in spiders are shown in Table 2. On Tryptone Soya agar (TSA), microbial counts ranged from 1.18 in *S. bipunctata* to 2.64 log cfu/g in *S. castanea*. On Triple Sugar Iron (TSI) agar, microbial counts were from 1.11 in *T. domestica* to 3.26 log cfu/g in *P. lunata*. On Blood agar (BA), microbial counts were from 1.18 in *S. thoracica* to 2.95 log cfu/g in *M. ferruginea*. On Anaerobic agar (AA), microbial counts ranged from 1.11 in *S. bipunctata* to 2.84 log cfu/g in *M. ferruginea*.

### 3.2. Isolated Microbial Genera and Microbial Species from Spider Specimens

Isolated genera and species are shown in Table 3. In total, 28 genera and 56 microbial species from spider specimens were isolated. The most abundant species were *Bacillus pumilus* (28 isolates) and *B. thuringensis* (28 isolates). The least isolated species were *Rhodotorula mucilaginosa* (one isolate), *Kocuria rhizophila* (two isolates), *Paenibacillus polymyxa* (two isolates), and *Staphylococcus equorum* (two isolates).

**Figure 1:**
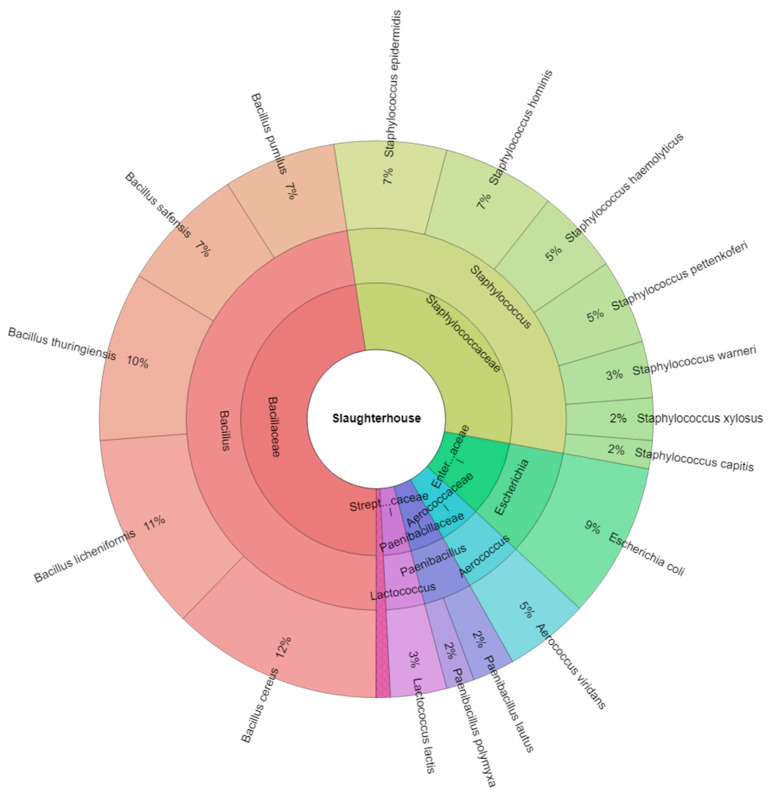
Krona chart. Percentual proportion of microbiota of arthropods originated from the slaughterhouse.

The composition of arthropod microbiota is shown in Figure 1. In total, 7 genera and 18 microbial species were isolated. The most abundant microbial genera were *Bacillus* (47.6%) and *Staphylococcus* (30.4%). For *Bacillus* spp., the most isolated species were *B. cereus* (12%) and *B. licheniformis* (11%), while for *Staphylococcus* spp. were *St. epidermidis* (7%) and *St. hominis* (7%).

**Figure 2:**
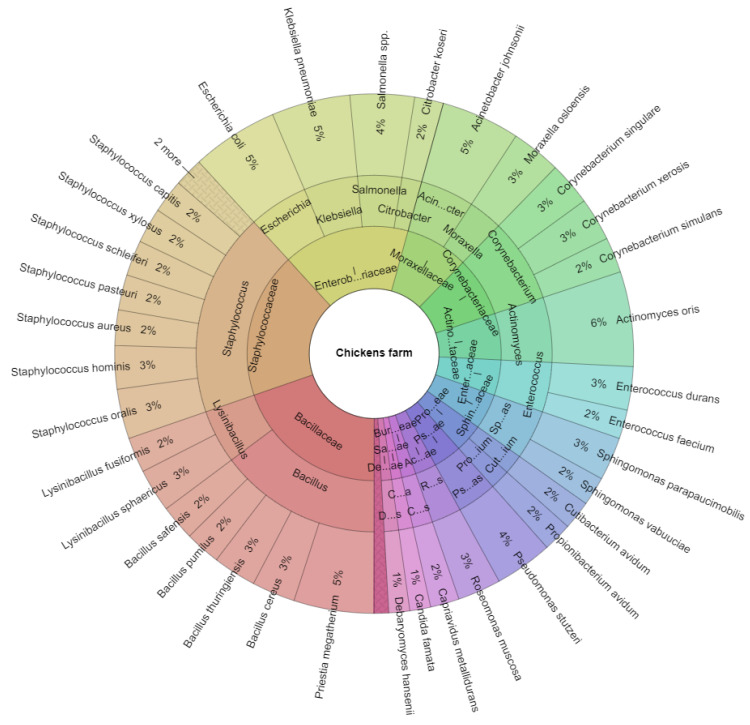
Krona chart. Percentual proportion of microbiota of arthropods originated from the chicken farm.

The composition of the microbiota of arthropods isolated from the chicken farm is shown in Figure 2, with a total of 20 genera and 38 microbial species isolated. The most isolated genera were *Staphylococcus* (18.5%) and *Bacillus* (14.5%). The most isolated species were *Actinomyces oris* (6%), *Escherichia coli*, and *Klebsiella pneumoniae* (5%).

**Figure 3:**
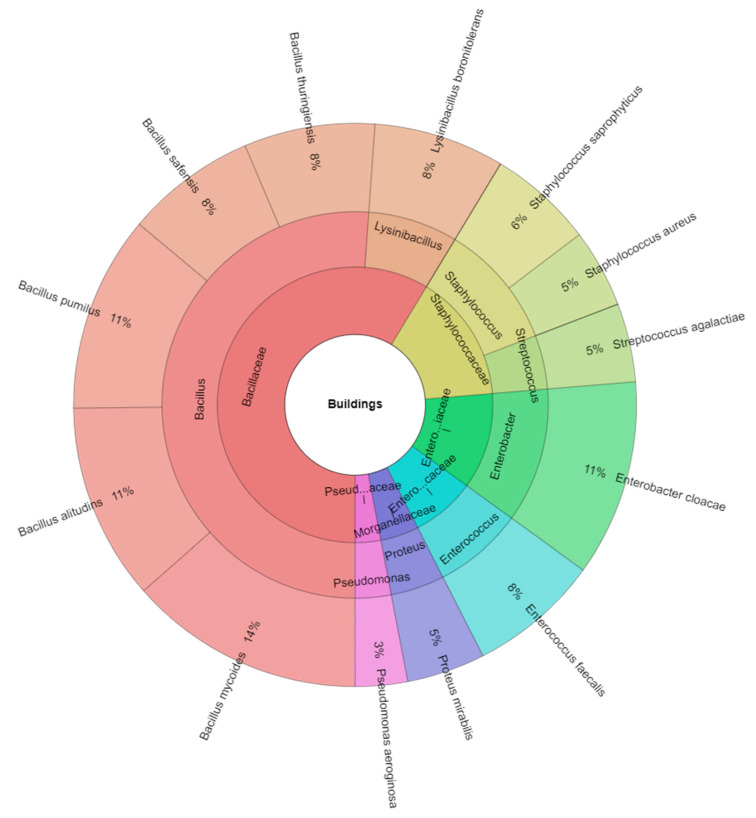
Krona chart. Percentual proportion of microbiota of arthropods isolated from buildings.

The microbial composition of arthropods microbiota in buildings is shown in Figure 3. In total, 7 genera and 13 microbial species were isolated. The most isolated genera were *Bacillus* (51.13%) and the most abundant species were *B. mycoides* (14%), *B. alitudins, B. pumilus*, and *E. cloacae* (11%).

### 3.3. Antibiotic Resistance of Isolated Microbial Species of Spiders

The antimicrobial resistance of isolated microorganisms from slaughterhouse is shown in Table 4. In total, 127 species isolated from the slaughterhouse were resistant to different antibiotics. Sensitivity to antibiotic resistance was found in 333 isolates.

Antibiotic resistance/sensitivity of microbiota isolated from the chicken farm is shown in Table 5. In total, 108 species isolated from the chicken farm were resistant to different antibiotics. Sensitivity to antibiotic resistance was found in 620 isolates.

Antibiotic resistance/sensitivity of microbiota isolated from buildings is shown in Table 6. In total, 114 species isolated from buildings were resistant to different antibiotics. Sensitivity to antibiotic resistance was found in 494 isolates.

## 4. Discussion

The microbiome of the individual animal is unique and reflects the life history and modulates behavior, the composition of the microbiota is essential in maintaining health and welfare [30,31,32]. Microbiota of arthropods was reported to be of importance in the dissemination of the pathogens of animals and human health importance and antimicrobial resistance genes. Reports on the isolation of the pathogens transferred by arthropods inhabiting premises for livestock and poultry production and the transfer of potentially virulent antimicrobial-resistant enterococci in pig operations confirm the importance of insects for maintenance of the pathogens and antimicrobial resistance genes within the agricultural environment [33]. This highlights the need for studies associated with arthropods microbiota and the heavily contaminated environment of the poultry farms, which is associated with a high stocking density of birds.

In the present study, the microbial counts were different for spider species and types of habitats. The microbial counts in our study were lower than in the study by Voloshyn et al. [34], who reported microbial counts of 3.18 log CFU/mL for *Escherichia coli* isolated from the surface of *Lithobius* sp. to 5.65 log CFU/mL for *Pseudomonas aeruginosa* isolated from the surface of *Fannia* sp.; also, the staphylococci were found to inhabiting the arthropods in high counts (3.91–5.61 log CFU/mL). Among the pathogenic bacteria, *Pseudomonas aeruginosa* and *Klebsiella pneumonia* were isolated. *Escherichia coli* was the most common microorganism on the external surface of arthropods.

Keiser et al. [9] studied the dominant microbiota of social spiders in spider cuticula and found similar microbial composition between the spiders, webs, and preys that may indicate that spiders themselves may enhance microbial transmission. This may explain the similarities between the composition of bacteriota that were identified in the present study.

As for humans and animals, arthropods harbor large microbial communities, which may exceed the numbers of organism’s cells of their hosts [35,36]. Moreover, the microbiota of certain arthropods was found to be very diverse, with multiple microbial families represented [37]. Different microorganisms were shown to be inhabiting the digestive tract and/or salivary glands of arthropods; subsequently, this microbiota primary may interact with vector-borne pathogens and affect their lifecycle. A study by Zhang et al. [38] revealed the presence of four microbial phyla, including Actinobacteria, Firmicutes, Fungi, and Proteobacteria, which were identified in all spider species. Proteobacteria was the most abundant phylum, while a total of 28 families and 58 species were identified [38]. Differences in the composition of microbiota between the spiders regarding their ecology and behavior were non-significant, and the microbiome of solitary spiders was characterized by low diversity [38,39,40]. The current research on the microbiota of spiders provides knowledge on the microbial composition of arachnoids.

*B. cereus*, *B. licheniformis*, *St. epidermidis*, and *St. hominis* were the most abundant microbial species originating from the slaughterhouse, while *A. oris*, *E. coli*, and *K. pneumoniae* were the most abundant species found in chicken farm samples. *B. mycoides*, *B. alitudins, B. pumilus*, and *E. cloacae* were associated with spiders obtained from the buildings. The ecological niche is found to pose significant impact on the microbiota of spiders. Spiders are colonized with diverse microbiota, including pathogens from the surrounding environment and feed, especially on carrion insects. The immune system of arthropods protects them against infections with pathogenic microorganisms [41,42,43]. Once their tissues are damaged, the microbiota may overcome external barrier and enter the deeper layer of tissues [44]. Thus, the spiders may acquire the pathogens from the surrounding environment and distribute them as a mechanical vector [45,46]. The composition of microbial communities differs between sites of the arachnoides. *Bacillus* spp. were not recovered from spider walks in contrast to body cavities such as the abdomen, while only *Kluyvera* and *Staphylococcus* spp. were isolated from spider walks. Diverse microbial communities on the chelicerae were reported to be the most and include *Pseudomonas*, *Rothia*, *Streptococcus*, and *Staphylococcus* spp. [47]. *Staphylococcus* spp. were recovered from *S. nobilis, A. similis*, and *E. atrica* with staphylococci species were recovered from *S. nobilis*. Among isolated species, some may pose public and environmental health implications. *S. epidermidis* is reported to cause severe conditions in susceptible individuals with clinical manifestations including bacteremia and septicemia, urinary tract infections, and endocarditis. Contamination of medicinal equipment may result in nosocomial sepsis. Additionally, other *Staphylococcus* species were identified as opportunistic human pathogens, which may be severe in immunocompromised hosts. Despite being a part of normal skin microbiota may cause an infection if the immune system functions are altered or there is a disbalance in the composition of normal microbiota that may lead to enhanced colonization [48,49,50].

*Bacillus*, *Paenibacillus*, *Pseudomonas*, and *Staphylococcus* spp. were identified in spiders in our study, and *Bacillus thuringiensis* was present in all samples. *B. thuringiensis* is a soil-dwelling microorganism, which is highly pathogenic for insects, and cases of human infection were reported. Among well-established pathogens of public health importance, *K. pneumniae, E. coli*, and *Sallmonella* spp. were found. The presence of *Salmonella*, *Bacillus*, *Staphylococcus*, and *Escherichia* species was reported in *Amaurobius similis*, *Eratigena atrica*, and *Steatoda nobilis*, which is in agreement with our results [51]. Those findings are important since not only show the evidence of possible transmission of pathogens to environment, humans, and animals, but also pose antimicrobial resistance threats. Resistance in *Salmonella* spp. to ciprofloxacin is alarming since it is used in humans for treatment of salmonellosis; therefore, the antimicrobial resistance in spiders is of concern.

Yeasts of *Candida*, *Debaryomyces*, and *Rhodotorula* were a part of spider’s microbiome in the present study. Recent research found cuticular antimicrobials as the first-line defense against infection and fungal growth and those antimicrobials were described in subsocial crab spiders [52], suggesting that cuticular immune-related properties could be at play [53,54,55,56].

The antimicrobial resistance of spider surface microbiome was identified in spiders sampled from all locations. The highest prevalence of resistant bacteria was found in the slaughterhouse (38%), followed by samples from buildings (23%) and chicken farm (7%). Spiders of *Latrodectus esperus* were recognized to transfer highly pathogenic and multidrug resistance bacteria, which may cause necrotic arachnidism, while first-line antibiotic treatment has been shown to be ineffective for the treatment of this infection [46]. Additionally, bites of *S. nobilis* may require antimicrobial treatment, especially for the medical staff affected [47]. In previous studies of *Steatoda nobilis* microbiota, *p. putida* was associated with resistance to three broad range antibiotics (amoxicillin, erythromycin, and cefoxitin), while *S. capitis* was multidrug-resistant and revealed antimicrobial resistance against a different class of antibiotics (gentamicin, tetracycline, and nalidixic acid) but *S. edaphicus* to gentamicin, chloramphenicol, and nalidixic acid. Resistance to tetracycline and chloramphenicol was reported in *S. capitis* and *S. edaphicus*, respectively. In terms of the identified resistances, resistance to nalidixic acid, erythromycin, cefoxitin, gentamicin, amoxycillin, colistin, tetracycline, and chloramphenicol was identified while all *S. nobilis* isolates were susceptible to ciprofloxacin [47].

Results of our study suggest that spiders of different locations may harbor similar microbial communities between different habitats. However, the spiders may transfer microorganisms between prey, predator, and the wider environment. Transgenerational transmission of symbiotic microorganisms is important for arthropods which may experience large-scale mortality events [57].

## 5. Conclusions

Spiders are among the most diverse and abundant predators in agroecosystems. External surfaces of spiders are inhabited by diverse microbiota, with Proteobacteria being the predominant phylum and *Bacillus* and *Staphylococcus* being the most abundant bacteria genera. Our study demonstrates that 14 spider species carried opportunistic pathogenic bacteria on their body surfaces that may result in the vector-borne transmission of different pathogens, including zoonoses. Multiresistance and resistance to antimicrobials important for human medicine were recognized in spider isolates that can provide evidence of their possible involvement in the dissemination of antimicrobial resistance. The present study could be a contribution to research on microbial compositions and antimicrobial resistance of their isolates with potential public and environmental health implications.

## Figures and Tables

**Table 1:** Identified spiders and their locations.

Location	Spider Species	Gender
Nitra City, 48°18′ N 18°05′ E, Slaughterhouse	1. *Pholcus alticeps* (Spassky, 1932)	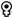
	2. *Pholcus alticeps* (Spassky, 1932)	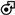
	3. *Pholcus alticeps* (Spassky, 1932)	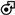
	4. *Pholcus alticeps* (Spassky, 1932)	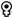
	5. *Pholcus alticeps* (Spassky, 1932)	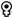
	6. *Steatoda triangulosa* (Walckenaer, 1802)	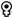
	7. *Steatoda triangulosa* (Walckenaer, 1802)	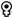
	8. *Pholcus alticeps* (Spassky, 1932)	juv.
Nové Zámky region, Jatov village, 48°10′ N 18°00′ E, house	9. *Steatoda bipunctata* (Linnaeus, 1758)	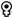
	*10. Steatoda bipunctata* (Linnaeus, 1758)	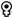
	*11. Scytodes thoracica* (Latreille, 1802)	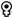
Nitra City, 48°18′ N 18°05′ E, apartment building	12. *Pholcus phalangioides* (Fuesslin, 1775)	juv.
	13. *Pholcus phalangioides* (Fuesslin, 1775)	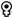
	14. *Pholcus phalangioides* (Fuesslin, 1775)	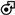
	15. *Pholcus phalangioides* (Fuesslin, 1775)	juv.
	16. *Pholcus phalangioides* (Fuesslin, 1775)	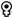
	17. *Pholcus phalangioides* (Fuesslin, 1775)	juv.
	*18. Pholcus phalangioides* (Fuesslin, 1775)	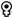
	19. *Pholcus phalangioides* (Fuesslin, 1775)	juv.
	20. *Pholcus phalangioides* (Fuesslin, 1775)	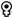
Nové Zámky region, Jatov village, 48°10′ N 18°00′ E, house	21. *Malthonica ferruginea* (Panzer, 1804)	
Nitra City, Street, 48°18′ N 18°05′ E, Student dormitory	22. *Steatoda triangulosa* (Walckenaer, 1802)	
	23. *Steatoda triangulosa* (Walckenaer, 1802)	
Nitra City, 48°18′ N 18°05′ E, SPU, building	24. *Pholcus phalangioides* (Fuesslin, 1775)	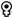
	25. *Pholcus phalangioides* (Fuesslin, 1775)	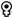
	26. *Pholcus phalangioides* (Fuesslin, 1775)	juv.
	27. *Pholcus phalangioides* (Fuesslin, 1775)	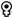
	28. *Pholcus phalangioides* (Fuesslin, 1775)	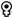
	29. *Pholcus phalangioides* (Fuesslin, 1775)	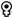
	30. *Steatoda triangulosa* (Walckenaer, 1802)	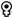
	31. *Steatoda triangulosa* (Walckenaer, 1802)	juv.
Nitra City, 48°18′ N 18°05′ E, apartment building	32. *Pholcus phalangioides* (Fuesslin, 1775)	juv.
	33. *Pholcus phalangioides* (Fuesslin, 1775)	juv.
	34. *Pholcus phalangioides* (Fuesslin, 1775)	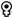
	35. *Pholcus phalangioides* (Fuesslin, 1775)	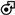
	36. *Steatoda triangulosa* (Walckenaer, 1802)	juv.
	37. *Steatoda triangulosa* (Walckenaer, 1802)	juv.
Nové Zámky region, Jatov village, 48°10′ N 18°00′ E, house	38. *Steatoda triangulosa* (Walckenaer, 1802)	juv.
	39. *Trochosa robusta* (Simon, 1876)	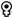
Nitra City, 48°18′ N 18°05′ E, Botanical Garden SPU	40. *Pardosa hortensis* (Thorell, 1872)	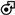
	41. *Pardosa hortensis* (Thorell, 1872)	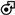
	42. *Pardosa hortensis* (Thorell, 1872)	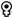
	43. *Pardosa hortensis* (Thorell, 1872)	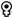
Veľký Lapáš Bodok, 48°17′24′′ S 18°11′09′′ V, chicken farm	44. *Parasteatoda tepidariorum* (C. L. Koch, 1841)	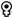
	45. *Parasteatoda tepidariorum* (C. L. Koch, 1841)	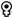
	46. *Parasteatoda tepidariorum* (C. L. Koch, 1841)	juv.
	47. *Pholcus phalangioides* (Fuesslin, 1775)	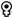
	48. *Pholcus phalangioides* (Fuesslin, 1775)	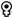
	49. *Parasteatoda tepidariorum* (C. L. Koch, 1841)	juv.
	50. *Steatoda bipunctata* (Linnaeus, 1758)	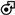
	51. *Steatoda bipunctata* (Linnaeus, 1758)	juv.
	52. *Steatoda bipunctata* (Linnaeus, 1758)	juv.
	53. *Steatoda bipunctata* (Linnaeus, 1758)	juv.
	54. *Steatoda bipunctata* (Linnaeus, 1758)	juv.
	55. *Steatoda bipunctata* (Linnaeus, 1758)	juv.
	56. *Steatoda bipunctata* (Linnaeus, 1758)	juv.
	57. *Steatoda bipunctata* (Linnaeus, 1758)	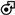
	58. *Steatoda bipunctata* (Linnaeus, 1758)	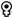
	59. *Steatoda bipunctata* (Linnaeus, 1758)	juv.
	60. *Steatoda bipunctata* (Linnaeus, 1758)	juv.
	61. *Pholcus alticeps* (Spassky, 1932)	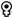
	62. *Pholcus alticeps* (Spassky, 1932)	juv.
	63. *Tegenaria domestica* (Clerck, 1757)	juv.
	64. *Tegenaria domestica* (Clerck, 1757)	juv.
	65. *Tegenaria domestica* (Clerck, 1757)	juv.
	66. *Tegenaria domestica* (Clerck, 1757)	juv.
	67. *Tegenaria domestica* (Clerck, 1757)	juv.
	68. *Parasteatoda lunata* (Clerck, 1757)	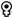
	69. *Parasteatoda tepidariorum* (C. L. Koch, 1841)	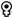
	70. *Steatoda triangulosa* (Walckenaer, 1802)	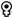
	71. *Steatoda castanea* (Clerck, 1757)	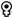
	72. *Salticus scenicus* (Clerck, 1757)	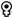
	73. *Nuctenea umbratica* (Clerck, 1757)	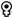
	74. *Steatoda triangulosa* (Walckenaer, 1802)	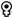
	75. *Pholcus phalangioides* (Fuesslin, 1775)	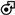
	76. *Pholcus phalangioides* (Fuesslin, 1775)	juv.
	77. *Pholcus phalangioides* (Fuesslin, 1775)	juv.
	78. *Steatoda bipunctata* (Linnaeus, 1758)	sub. 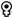
	79. *Tegenaria domestica* (Clerck, 1757)	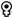
	80. *Steatoda bipunctata* (Linnaeus, 1758)	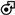
	81. *Steatoda bipunctata* (Linnaeus, 1758)	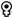
	82. *Steatoda triangulosa* (Walckenaer, 1802)	juv.
	83. *Parasteatoda tepidariorum* (C. L. Koch, 1841)	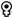
	84. *Steatoda bipunctata* (Linnaeus, 1758)	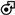
	85. *Steatoda bipunctata* (Linnaeus, 1758)	juv.
	86. *Steatoda bipunctata* (Linnaeus, 1758)	juv.
	87. *Steatoda bipunctata* (Linnaeus, 1758)	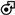
	88. *Steatoda bipunctata* (Linnaeus, 1758)	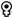
	89. *Steatoda bipunctata* (Linnaeus, 1758)	juv.
	90. *Steatoda bipunctata* (Linnaeus, 1758)	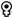
	91. *Steatoda bipunctata* (Linnaeus, 1758)	juv.
	92. *Pholcus phalangioides* (Fuesslin, 1775)	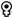
	93. *Pholcus phalangioides* (Fuesslin, 1775)	juv.
	94. *Pholcus phalangioides* (Fuesslin, 1775)	juv.
	95. *Pholcus phalangioides* (Fuesslin, 1775)	juv.
	96. *Pholcus phalangioides* (Fuesslin, 1775)	juv.
	97. *Pholcus phalangioides* (Fuesslin, 1775)	juv.
	98. *Pholcus phalangioides* (Fuesslin, 1775)	juv.
	99. *Pholcus phalangioides* (Fuesslin, 1775)	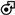
	100. *Pholcus alticeps* (Spassky, 1932)	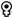
	101. *Tegenaria domestica* (Clerck, 1757)	juv.
	102. *Tegenaria domestica* (Clerck, 1757)	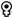

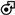
—male; 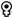
—female; juv.—juvenile.

**Table 2:** Microbial counts of spiders on individual agar (mean ± sd, in log cfu/g).

Spider/Agar	TSA	TSI	BA	AA
*Malthonica ferruginea*	2.37 ± 0.03 ^a^	2.81 ± 0.03 ^b^	2.95 ± 0.02 ^c^	2.84 ± 0.05 ^d^
*Nuctenea umbratica*	1.48 ± 0.03 ^a^	1.26 ± 0.06 ^b^	1.55 ± 0.10 ^c^	1.50 ± 0.05 ^d^
*Parasteatoda lunata*	3.39 ± 0.12 ^a^	3.26 ± 0.06 ^b^	2.84 ± 0.06 ^c^	2.45 ± 0.08 ^d^
*Parasteatoda tepidariorum*	1.38 ± 0.19 ^a^	1.43 ± 0.10 ^b^	1.34 ± 0.12 ^c^	1.44 ± 0.08 ^d^
*Pardosa hortensis*	1.83 ± 0.05 ^a^	1.57 ± 0.09 ^b^	1.80 ± 0.09 ^c^	1.53 ± 0.07 ^d^
*Pholcus alticeps*	1.34 ± 0.09 ^a^	1.28 ± 0.04 ^b^	2.58 ± 0.06 ^c^	1.49 ± 0.04 ^d^
*Pholcus phalangioides*	1.22 ± 0.06 ^a^	1.28 ± 0.05 ^b^	1.19 ± 0.02 ^c^	1.27 ± 0.09 ^d^
*Salticus scenicus*	2.30 ± 0.05 ^a^	2.31 ± 0.16 ^b^	2.34 ± 0.08 ^c^	2.27 ± 0.09 ^d^
*Scytodes thoracica*	1.29 ± 0.06 ^a^	1.28 ± 0.03 ^b^	1.18 ± 0.03 ^c^	1.45 ± 0.08 ^d^
*Steatoda bipunctata*	1.18 ± 0.07 ^a^	1.19 ± 0.05 ^b^	1.22 ± 0.06 ^c^	1.11 ± 0.06 ^d^
*Steatoda castanea*	2.64 ± 0.21 ^a^	2.30 ± 0.06 ^b^	2.72 ± 0.06 ^c^	2.28 ± 0.16 ^d^
*Steatoda triangulosa*	1.43 ± 0.06 ^a^	1.30 ± 0.06 ^b^	1.79 ± 0.07 ^c^	1.43 ± 0.08 ^d^
*Tegenaria domestica*	1.19 ± 0.05 ^a^	1.11 ± 0.06 ^b^	1.27 ± 0.08 ^c^	1.29 ± 0.12 ^d^
*Trochosa robusta*	2.46 ± 0.11 ^a^	2.20 ± 0.03 ^b^	2.46 ± 0.09 ^c^	2.21 ± 0.14 ^d^

TSA—Tryptone Soya Agar; TSI—Triple Sugar Agar; BA—blood agar; AA—Anaerobic Agar; ^a^ Differences between the microbial counts on TSA agar between different spider species were significant (*p* < 0.01). ^b^ Differences between the microbial counts on TSI agar between different spider species were significant (*p* < 0.01). ^c^ Differences between the microbial counts on BA agar between different spider species were significant (*p* < 0.01). ^d^ Differences between the microbial counts on AA agar between different spider species were significant (*p* < 0.01).

**Table 3:** Microbial genera and microbial species of arthropods.

Phylum	Taxa/Spider Specimens	Slaughterhouse	Chicken Farm	Buildings
Proteobacteria	***Acinetobacter***			
	*Acinetobacter johnsonii*	-	11	-
Actinobacteria	***Actinomyces***			
	*Actinomyces oris*	-	13	-
Firmicutes	***Aerococcus***			
	*Aerococcus viridans*	6	-	-
Firmicutes	***Bacillus***			
	*Bacillus alitudins*	-	-	15
	*Bacillus cereus*	15	6	-
	*Bacillus licheniformis*	14	-	-
	*Bacillus megatherium*	-	10	-
	*Bacillus mycoides*	-	-	18
	*Bacillus pumilus*	8	5	15
	*Bacillus safensis*	9	5	10
	*Bacillus thuringiensis*	12	6	10
Fungi	***Candida***			
	*Candida famata*	-	3	-
Proteobacteria	***Capriavidus***			
	*Capriavidus metallidurans*	-	4	-
Proteobacteria	***Citrobacter***			
	*Citrobacter koseri*	-	4	-
Actinobacteria	***Corynebacterium***			
	*Corynebacterium simulans*	-	5	-
	*Corynebacterium singulare*	-	6	-
	*Corynebacterium xerosis*	-	6	-
Actinobacteria	***Cutibacterium***			
	*Cutibacterium avidum*	-	4	-
Fungi	***Debaryomyces***			
	*Debaryomyces hansenii*	-	3	-
Proteobacteria	***Enterobacter***			
	*Enterobacter cloacae*	-	-	15
Firmicutes	***Enterococcus***			
	*Enterococcus durans*	-	6	-
	*Enterococcus faecalis*	-	-	10
	*Enterococcus faecium*	-	4	-
Proteobacteria	***Escherichia***			
	*Escherichia coli*	11	12	-
Proteobacteria	***Klebsiella***			
	*Klebsiella pneumoniae*	-	11	-
Actinobacteria	***Kocuria***			
	*Kocuria rhizophila*	-	2	-
Firmicutes	***Lactococcus***			
	*Lactococcus lactis*	4	-	-
Firmicutes	***Lysinibacillus***			
	*Lysinibacillus boronitolerans*	-	-	10
	*Lysinibacillus fusiformis*	-	5	-
	*Lysinibacillus sphaericus*	-	6	-
Proteobacteria	***Moraxella***			
	*Moraxella osloensis*	-	7	-
Firmicutes	***Paenibacillus***			
	*Paenibacillus lautus*	3	-	-
	*Paenibacillus polymyxa*	2	-	-
Actinobacteria	***Propionibacterium***			
	*Propionibacterium avidum*	-	4	-
Proteobacteria	***Proteus***			
	*Proteus mirabilis*	-	-	6
Proteobacteria	***Pseudomonas***			
	*Pseudomonas aeroginosa*	-	-	4
	*Pseudomonas stutzeri*	-	8	-
Fungi	***Rhodotorula***			
	*Rhodotorula mucilaginosa*	1	-	-
Proteobacteria	***Roseomonas***			
	*Roseomonas muscosa*	-	6	-
Proteobacteria	***Salmonella***			
	*Salmonella* spp.	-	9	-
Proteobacteria	***Sphingomonas***			
	*Sphingomonas parapaucimobilis*	-	6	-
	*Sphingomonas vabuuciae*	-	4	-
Firmicutes	***Staphylococcus***			
	*Staphylococcus aureus*	-	5	6
	*Staphylococcus capitis*	2	4	-
	*Staphylococcus epidermidis*	8	2	-
	*Staphylococcus equorum*	-	2	-
	*Staphylococcus haemolyticus*	6	-	-
	*Staphylococcus hominis*	8	6	-
	*Staphylococcus oralis*	-	7	-
	*Staphylococcus pasteuri*	-	5	-
	*Staphylococcus pettenkoferi*	6	-	-
	*Staphylococcus saprophyticus*	-	-	8
	*Staphylococcus schleiferi*	-	5	-
	*Staphylococcus warneri*	4	-	-
	*Staphylococcus xylosus*	3	5	-
Firmicutes	***Streptococcus***			
	*Streptococcus agalactiae*	-	-	6
	**Total isolates**	**122**	**222**	**133**

**Table 4:** Antibiotic resistance in spider’s microbiota from the slaughterhouse.

Isolated Species	Antibiotic (R/S)				
	IPM	MEM	CIP	VA	LZD
*Aerococcus viridans*	ND	ND	ND	ND	ND
*Bacillus cereus*	2/13	2/13	3/12	5/10	0/15
*Bacillus licheniformis*	4//10	5/9	10/4	4/10	3/14
*Bacillus pumilus*	0/8	1/7	2/6	0/8	1/7
*Bacillus safensis*	0/9	1/8	1/8	1/8	3/6
*Bacillus thuringiensis*	2/10	3/9	2/10	1/11	0/12
	**IPM**	**MEM**	**CIP**	**TOB**	**C**
*Escherichia coli*	10/1	1/10	5/6	6/5	3/8
*Lactococcus lactis*	ND	ND	ND	ND	ND
*Paenibacillus lautus*	ND	ND	ND	ND	ND
*Paenibacillus polymyxa*	ND	ND	ND	ND	ND
*Rhodotorula mucilaginosa*	ND	ND	ND	ND	ND
	**CIP**	**NOR**	**AK**	**TOB**	**TGC**
*Staphylococcus capitis*	0/2	0/2	0/2	0/2	1/1
*Staphylococcus epidermidis*	2/6	1/7	2/6	3/5	4/4
*Staphylococcus haemolyticus*	5/1	2/4	3/3	0/6	1/5
*Staphylococcus hominis*	0/8	1/7	2/6	1/7	3/5
*Staphylococcus pettenkoferi*	0/6	2/4	1/5	2/4	1/5
*Staphylococcus warneri*	1/3	2/2	0/4	3/1	0/4
*Staphylococcus xylosus*	2/1	1/2	0/3	0/3	0/3
Total	28/78	22/84	31/75	26/80	20/86

R—resistant; S—sensitive; ND—not determined; IPM—imipenem; MEM—meropenem; CIP—ciprofloxacin; VA—vancomycin; LZD—linezolid; TOB—tobramycin; TGC—tigecycline; AK—amikacin; NOR—norfloxacin; TE—tetracycline.

**Table 5:** Antibiotic resistance in spider’s microbiota from the chicken farm.

Isolated Species	Antibiotic (R/S)				
	IPM	MEM	CIP	VA	LZD
*Acinetobacter johnsonii*	ND	ND	ND	ND	ND
*Actinomyces oris*	ND	ND	ND	ND	ND
*Bacillus cereus*	1/5	2/4	0/6	3/3	1/5
*Priestia megatherium*	0/10	1/9	0/10	1/9	2/8
*Bacillus pumilus*	0/5	0/5	0/5	0/5	1/4
*Bacillus safensis*	0/5	1/4	0/5	1/4	1/4
*Bacillus thuringiensis*	0/6	1/5	0/6	1/5	1/5
*Candida famata*	ND	ND	ND	ND	ND
*Capriavidus metallidurans*	ND	ND	ND	ND	ND
	**IPM**	**MEM**	**CIP**	**TOB**	**C**
*Citrobacter koseri*	0/4	1/3	0/4	1/3	2/2
*Escherichia coli*	2/10	3/9	2/10	5/7	2/10
*Klebsiella pneumoniae*	1/10	2/9	2/9	0/11	1/10
*Salmonella* spp.	0/9	1/8	1/8	1/8	0/9
	**CIP**	**VA**	**TE**	**LZD**	**RD**
*Corynebacterium simulans*	0/5	1/4	0/5	0//5	1/4
*Corynebacterium singulare*	1/5	0/6	2/4	0/6	1/5
*Corynebacterium xerosis*	0/6	1/5	0/6	0/6	0/6
	**MEM**	**VA**			
*Cutibacterium avidum*	0/4	0/4	-	-	-
*Debaryomyces hansenii*	ND	ND	ND	ND	ND
	**IMP**	**CIP**	**VA**	**TGC**	**LZD**
*Enterococcus durans*	2/4	3/3	2/4	6/0	1/5
*Enterococcus faecium*	1/3	2/2	3/1	1/3	2/2
*Kocuria rhizophila*	ND	ND	ND	ND	ND
*Lysinibacillus fusiformis*	ND	ND	ND	ND	ND
*Lysinibacillus sphaericus*	ND	ND	ND	ND	ND
*Moraxella osloensis*	ND	ND	ND	ND	ND
	**IMP**	**MEM**	**CIP**	**TOB**	**AK**
*Pseudomonas stutzeri*	1/7	2/6	0/8	0/8	0/8
*Propionibacterium avidum*	ND	ND	ND	ND	ND
*Roseomonas muscosa*	ND	ND	ND	ND	ND
*Sphingomonas parapaucimobilis*	ND	ND	ND	ND	ND
*Sphingomonas vabuuciae*	ND	ND	ND	ND	ND
	**CIP**	**NOR**	**AK**	**TOB**	**TGC**
*Staphylococcus aureus*	1/4	0/5	0/5	2/3	1/4
*Staphylococcus capitis*	1/3	1/3	0/4	0/4	1/3
*Staphylococcus epidermidis*	0/2	0/2	0/2	0/2	0/2
*Staphylococcus equorum*	0/2	0/2	0/2	0/2	0/2
*Staphylococcus hominis*	1/5	1/5	2/4	0/6	0/6
*Staphylococcus oralis*	2/5	0/7	1/6	2/5	1/6
*Staphylococcus pasteuri*	1/4	0/5	0/5	1/4	1/4
*Staphylococcus schleiferi*	0/5	0/5	1/4	1/4	1/4
*Staphylococcus xylosus*	0/5	0/5	0/5	2/3	3/2
Total	15/133	23/125	16/128	29/115	25/119

R—resistant; S—sensitive; ND—not determined; IPM—imipenem; MEM—meropenem; CIP—ciprofloxacin; VA—vancomycin; LZD—linezolid; TOB—tobramycin; TGC—tigecycline; AK—amikacin; NOR—norfloxacin; TE—tetracycline; RD—rifampicin.

**Table 6:** Antibiotic resistance in spider’s microbiota from the buildings.

Isolated Species	Antibiotic (R/S)				
	IPM	MEM	CIP	VA	LZD
*Bacillus alitudins*	5/10	2/13	0/15	5/10	6/9
*Bacillus mycoides*	2/16	2/16	6/12	0/18	3/15
*Bacillus pumilus*	2/8	3/7	4/6	0/10	1/9
*Bacillus safensis*	2/8	2/8	0/10	5/5	6/4
*Bacillus thuringiensis*	2/8	3/7	5/5	0/10	4/6
	**IPM**	**MEM**	**CIP**	**TOB**	**C**
*Enterobacter cloacae*	0/15	0/15	0//15	0/15	0/15
*Proteus mirabilis*	0/6	0/6	0/6	0/6	0/6
	**IMP**	**CIP**	**VA**	**TGC**	**LZD**
*Enterococcus faecalis*	1/9	2/8	5/5	0/10	1/9
*Lysinibacillus boronitolerans*	ND	ND	ND	ND	ND
	**IMP**	**MEM**	**CIP**	**TOB**	**AK**
*Pseudomonas aeroginosa*	0/4	1/3	0/4	0/4	0/4
	**CIP**	**NOR**	**AK**	**TOB**	**TGC**
*Staphylococcus aureus*	0/6	1/5	2/4	0/6	0/6
*Staphylococcus saprophyticus*	1/7	1/7	0/8	0/8	2/6
	**VA**	**TGC**	**LZD**	**C**	**TE**
*Streptococcus agalactiae*	5/1	1/5	2/4	0/6	1/5
Total	20/98	18/100	24/94	10/108	24/94

R—resistant; S—sensitive; ND—not determined; IPM—imipenem; MEM—meropenem; CIP—ciprofloxacin; VA—vancomycin; LZD—linezolid; TOB—tobramycin; TGC—tigecycline; AK—amikacin; NOR—norfloxacin; TE—tetracycline.

## Data Availability

No new data were created or analyzed in this study. Data sharing is not applicable to this article.
